# Does Tropical Forest Fragmentation Increase Long-Term Variability of Butterfly Communities?

**DOI:** 10.1371/journal.pone.0009534

**Published:** 2010-03-10

**Authors:** Allison K. Leidner, Nick M. Haddad, Thomas E. Lovejoy

**Affiliations:** 1 Department of Biology, North Carolina State University, Raleigh, North Carolina, United States of America; 2 H. John Heinz III Center for Science, Economics and the Environment, Washington, District of Columbia, United States of America; 3 Biological Dynamics of Forest Fragments Project, Instituto Nacional de Pesquisas da Amazônia and Smithsonian Tropical Research Institute, Manaus, Amazonas, Brazil; 4 Department of Environmental Science and Policy, George Mason University, Fairfax, Virginia, United States of America; University of Hull, United Kingdom

## Abstract

Habitat fragmentation is a major driver of biodiversity loss. Yet, the overall effects of fragmentation on biodiversity may be obscured by differences in responses among species. These opposing responses to fragmentation may be manifest in higher variability in species richness and abundance (termed hyperdynamism), and in predictable changes in community composition. We tested whether forest fragmentation causes long-term hyperdynamism in butterfly communities, a taxon that naturally displays large variations in species richness and community composition. Using a dataset from an experimentally fragmented landscape in the central Amazon that spanned 11 years, we evaluated the effect of fragmentation on changes in species richness and community composition through time. Overall, adjusted species richness (adjusted for survey duration) did not differ between fragmented forest and intact forest. However, spatial and temporal variation of adjusted species richness was significantly higher in fragmented forests relative to intact forest. This variation was associated with changes in butterfly community composition, specifically lower proportions of understory shade species and higher proportions of edge species in fragmented forest. Analysis of rarefied species richness, estimated using indices of butterfly abundance, showed no differences between fragmented and intact forest plots in spatial or temporal variation. These results do not contradict the results from adjusted species richness, but rather suggest that higher variability in butterfly adjusted species richness may be explained by changes in butterfly abundance. Combined, these results indicate that butterfly communities in fragmented tropical forests are more variable than in intact forest, and that the natural variability of butterflies was not a buffer against the effects of fragmentation on community dynamics.

## Introduction

Theoretical and empirical research provides overwhelming evidence that habitat fragmentation reduces biodiversity [1], [2]. Yet, in some cases, detrimental effects of fragmentation on diversity may be obscured by variation in responses across taxa and through time [reviewed in 3], [4], [5]. For example, fragmentation has been found to positively, negatively, and neutrally affect species richness for species within the same study [6], [7], [8], among higher taxa [9], [10], and even within genera [11]. Using a long-term dataset on a diverse taxon, butterflies, we test how fragmentation affects community composition and the long-term dynamics of species richness.

One potential explanation for contrasting results among studies and taxa is that fragmentation may increase variability of community diversity or composition. Laurance [12] suggests that hyperdynamism, an “increase in the frequency and/or amplitude of population, community, and landscape dynamics in fragmented habitats,” can lead to a wide array of changes in biodiversity, the effects of which depend on the time since fragmentation and stochastic demographic and environmental factors. Some long-term fragmentation studies have tested for hyperdynamism. A 22-year study on tropical trees showed that species richness was hyperdynamic in fragmented forests relative to intact forests, even though fragmentation did not reduce average tree species richness [13]. In a two-decade study in eastern U.S. forests, species richness of breeding birds had higher temporal variability of species richness in forest fragments [14].

There are at least two potential explanations for increased variability in species richness within fragmented landscapes. First, resident populations may exhibit increased variability in abundance after fragmentation. Second, fragmentation may change community composition, favoring species that fluctuate more in their population sizes. For example, in the same experimental landscape as the tropical tree study, beetles that were rare or had naturally fluctuating abundances were more likely than species with stable abundances to persist in forest fragments a decade after fragmentation [11].

Insects, the most diverse higher taxon [15], [16], are naturally subject to population surges and crashes, and anthropogenic disturbances can exacerbate these dynamics [17], [18], [19]. Insects display variable population dynamics in part because they have the capacity to closely follow changes in the environment, such as weather and resource availability [20]. Consequently, hyperdynamism caused by habitat fragmentation may be less likely in insect communities than in long-lived and low-growth-rate taxa, such as birds, mammals, and trees. If fragmentation does cause hyperdynamism, increased variability in abundance would be expected to increase extinction risk [20], [21]. Insects, however, may again be an exception, because their natural fluctuations in abundance could buffer them against extinction, even when fragmentation increases population variability. Empirical evidence is mixed regarding the effects of fragmentation on rates of insect extinction relative to other taxa. The extinction rate of tropical butterflies in fragmented forests in Singapore was similar to that of plants and mammals, but higher than that of birds [22]. An analysis in Great Britain found that the local extinction rate of butterflies was higher than birds or plants [23]. These results suggest that if fragmentation results in hyperdynamism, it would negatively affect insects.

Here, we test the long-term effects of forest fragmentation on the dynamics of species richness and community composition of butterflies. We analyzed a dataset on butterfly species richness that spans 11 years from an experimentally fragmented landscape in the central Amazon. Rather than comparing forest fragments to continuous forest at a single point in time, we evaluated the effects of fragmentation on changes in species richness through time, focusing on three main questions:

1. Does habitat fragmentation reduce species richness, and do changes persist over time?
2. Does fragmentation increase spatial and temporal variation in species richness?
3. Does fragmentation alter butterfly community composition, and how might differences in community composition impact variability in species richness?

## Methods

### Study System

The Biological Dynamics of Forest Fragments Project (BDFFP) is a 1,000 km^2^ experimental landscape located in the central Amazon, approximately 80 km north of Manaus, Brazil. Starting in the early 1980s, tropical forest was cleared to create five 1 ha, four 10 ha, and two 100 ha forest fragments (referred to as fragmented forest plots). The same number of plots, in matching sizes, was established in nearby continuous forest (referred to as intact forest plots). Initially, the matrix surrounding forest fragments was maintained as cattle pasture, but over time some pastures were abandoned and subsequently invaded by shrubs and secondary forest species. A detailed description of the BDFPP experiment, including a map and plot history, can be found in Laurance and Bierregaard [24] and Laurance et al [7].

The data we analyzed were collected during butterfly surveys in the fragmented and intact forest plots between 1980–1986 and in 1991 (Figure S1). Extensive details on survey methodology are described in Brown and Hutchings [25]. Here, we provide a brief overview. In each plot during each survey period, observers recorded the identity and abundance (estimated to the nearest power of two) of each butterfly species (excluding skippers). Butterflies were identified visually with binoculars and a field key. In cases where visual identification was not possible, observers caught butterflies in hand nets for closer inspection. When weather was cloudy, the number of observer hours was increased.

Surveys were typically conducted on one day per year. Because of this, we restricted all of our analyses to data from one day each year. In the occasional years when there were additional surveys, we chose surveys across plots that were closest in time of year and observer hours (typically seven hours). We did not pool surveys for a given plot within a year because multiple surveys within a year were rare and incomplete, and because of high species turnover across seasons (see Text S1, Figure S2). Since the fragmented forest plots were isolated in different years, we analyzed data from fragmented forest plots based on time since fragmentation, rather than calendar year (year 1  =  year plot was fragmented). This was more biologically meaningful than calendar year, and accounted for temporary changes in species richness that are often observed immediately following fragmentation [3]. Because forest fragments were isolated in different years, our use of time since fragmentation allowed us to avoid a potentially confounding interaction between fragment age and the weather conditions of a particular year. We also conducted analyses showing that calendar year was not important in determining species richness in fragmented plots. We used analysis of variance (ANOVA) to test the importance of survey duration, plot identity, plot size, and survey year. In fragmented forest plots, we also tested for the effect of year since fragmentation on species richness. For both fragmented and intact forest plots, only survey duration was significant in predicting species richness (Table S1 and Table S2).

### Species Richness

As survey duration varied by plot and year (Figure S1), it was impossible to compare raw counts of species richness through time or across fragmentation status. Consequently, we used regression analysis to remove the effect of survey duration on species richness (henceforth termed “adjusted species richness”). Separately for fragmented and intact forest plots, we regressed observed species richness against observer hours. In cases where the regression was significant at *p*<0.100, we calculated adjusted species richness (*asr*) based on the slope of the linear regression using the formula
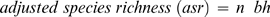
where *n*  =  number of species observed in the plot, *b*  =  slope of regression line, and *h*  =  number of hours the plot was surveyed −7 (the typical survey length in hours). For intact forest plots, size was not significant in determining species richness (Table S1), so we combined all plot sizes for the regression. Survey duration (hours) was significantly, positively related to species richness in intact forest plots (*t = *4.686, *p*<0.001, *R^2^* = 0.366, *n = *40, *b* = 4.256, 95% CI: 2.417–6.095, Figure S3), and was used to compute adjusted species richness. For fragmented forest plots, plot size was significant in determining species richness, so we separated the subsequent analyses of survey duration based on plot size (Table S2, Figure S4). For 1 ha fragmented forest plots, the regression was not significant, and we therefore did not adjust values of species richness (*t = *1.616, *p* = 0.121, *R^2^* = 0.111, *n = *23, *b* = 4.815, 95% CI: −1.382–11.012, Figure S4B). For 10 ha fragmented forest plots, the regression was significant (*t = *3.659, *p* = 0.002, *R^2^* = 0.454, *n = *18, *b* = 7.065, 95% CI: 2.961–11.168, Figure S4C), as it was for 100 ha fragmented forest plots (*t = *2.095, *p* = 0.090, *R^2^* = 0.467, *n = *7, *b* = 6.925, 95% CI: −1.574–15.425, Figure S4D).

We used ANOVA to test for effects of log-transformed plot size, fragmentation status (fragmented or intact), and their interaction on the adjusted species richness for each plot averaged across years. We then conducted analyses for each plot size, and compared the values of adjusted species richness post-fragmentation to average adjusted species richness in intact forest. We used a Dunnett's test to compare adjusted species richness of fragmented plots in each year after fragmentation to adjusted species richness of intact forest. Dunnett's test is a post-hoc test that accounts for multiple comparisons. It is more powerful than a Bonferroni test because the treatments are only compared to the control (intact forest), as opposed to every possible pairwise comparison.

### Temporal Variation (within Plots)

To test for the effects of fragmentation on temporal variation in adjusted species richness, we measured the average change in adjusted species richness within a plot over time. For each plot, we measured the change in adjusted species richness between two surveys using the formula:
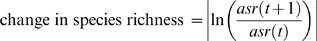
where *asr(t)* is the value of adjusted species richness during year *t* [based on 18]. So as not to average out changes in richness across several years, we only calculated this metric when the surveys of a given plot were separated by one year. Within each plot, we took the average of all of the individual changes. We then used a *t*-test (assuming unequal variance) to compare the average change in adjusted species richness between fragmented and intact plots. This measure is correlated with, but not identical to a more common measure of temporal variability, the coefficient of variation (*cv*). We did not use *cv* because 1) changes in species richness through time were not linear, so could not easily be detrended, and 2) we only wanted to examine changes in species richness from year-to-year.

### Spatial Variation (among Plots)

We measured spatial variation by examining the difference in adjusted species richness between plots within a given calendar year (intact forest plots) or number of years since fragmentation (fragmented forest plots). Separately, for each year in the intact and fragmented forest plots, we calculated the coefficient of variation (*cv*) of adjusted species richness (*cv* is appropriate in this case because trends are not relevant). We then averaged the values across all intact forest plots and fragmented forest plots. We analyzed the difference between fragmented and intact forest plots using *t*-tests and regressions to account for plot size (where *n*>2 for 1 ha and 10 ha plots, and *n = *2 for 100 ha plots).

### Effects of Abundance on Diversity

As species richness and species extinction rates depend on abundance, we used the only abundance data available, estimated to the nearest power of two (1, 2, 4, 8, 16, etc.), to calculate rarified species richness using Primer (Plymouth Routines in Multivariate Ecological Research, v.6). Rarefaction controls for the effects of butterfly abundance on their diversity [26]. Using rarefied species richness, we then recalculated temporal and spatial variation using the same methods as described above. Data based on indices of abundance are not the preferred type of data to use for rarefaction analysis, but we present the results because these were the only abundance data available. Consequently, the inferences we could draw from these indices of abundance were limited. Yet, they complement our analysis of adjusted species richness, allow a test of one potential mechanism driving variability, and suggest future avenues of research.

### Community Composition

We analyzed community composition based on the habitat association of species present in fragmented and intact forests. Previous analysis [25] suggested that a one-day survey of a given plot yielded 25–50% of the species actually present. This incomplete sampling resulted in artificially high species turnover rates between surveys, even when surveys of a given plot were separated by only a few days. Consequently, analyses based on similarity indices or ordination techniques could lead to spurious conclusions. We instead used percent composition, thus bypassing the problems associated with the abundance data and with the differences in survey effort between plots.

We grouped butterflies into the same four habitat association categories as Brown and Hutchings [25]: canopy and clearing species, understory sun species (species associated with light gaps), edge species, and understory shade species (species associated with closed canopy forest). For each plot we averaged the proportion composition for each habitat association across all surveys, as there was no clear temporal trend in composition. We then used ANOVA to examine the effects of fragmentation status (fragmented vs. intact), plot size, and their interaction on the proportion of species in each habitat association. Proportions were arcsine transformed.

## Results

We analyzed data from 40 butterfly surveys of 11 intact forest plots and 48 butterfly surveys of 11 fragmented forest plots. A total of 414 butterfly species was observed.

### Fragmentation and Adjusted Species Richness

**Figure 1:**
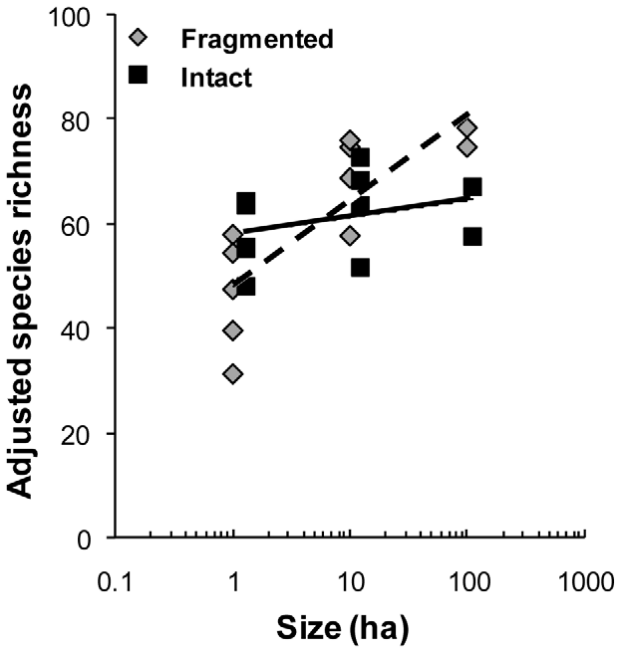
Relationship between size and species richness in fragmented and intact forest plots. Values of adjusted species richness were regressed against plot size for intact plots (solid squares, solid regression line) and fragmented plots (gray diamonds, dashed regression line). For fragmented plots, larger plots have more species. Intact plots are offset for visual emphasis only.

Log-transformed plot size, and the interaction between size and fragmentation status, were significant predictors of adjusted species richness (overall model F_3,18_ = 7.810, *p = *0.002; size, *p*<0.001; size*fragmentation status, *p* = 0.015, Figure 1). However, fragmentation status itself did not influence adjusted species richness (*p* = 0.865), as smaller fragmented plots had fewer species than intact plots, but larger fragmented plots had more species than intact plots (Figure 1).

**Figure 2:**
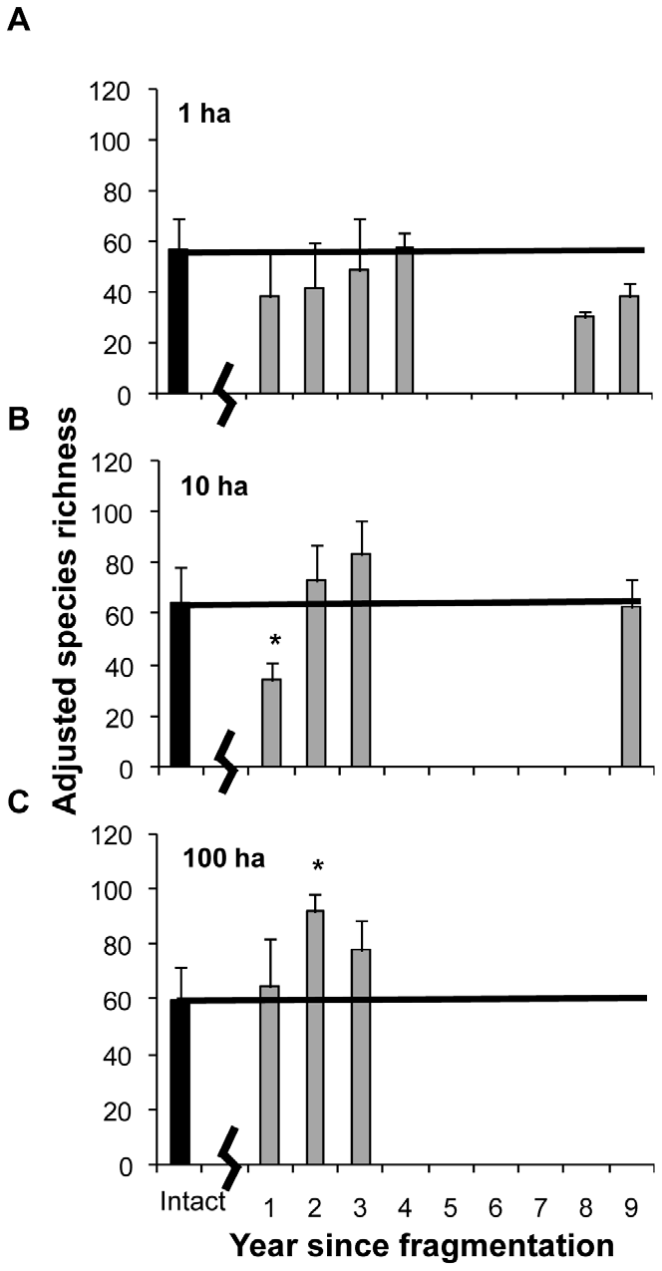
Comparison of species richness in intact forest plots to fragmented forest plots at various time points post-fragmentation. Adjusted species richness (+SD) for intact plots (black bars) and fragmented plots (gray bars) for (A) 1 ha plots, (B) 10 ha plots, and (C) 100 ha plots. Data from intact plots are combined across all years. Bars with asterisks indicate time points with a significant difference (*p≤*0.05) in species richness as compared to intact forest plots, using a post-hoc Dunnett's test.

Comparing intact forest plots to fragmented forest plots based on time since fragmentation showed an inconsistent response of adjusted species richness to fragmentation. In 1-ha plots, there was no difference in adjusted species richness between intact forest plots and fragmented forest plots at any time post-fragmentation (F_6,32_ = 2.389, *p* = 0.508, Figure 2A). In the 10-ha plots, time since fragmentation was a significant factor affecting adjusted species richness (F_4,21_ = 6.091, *p = *0.002, Figure 2B), as fragmented forest plots one year after fragmentation had lower adjusted species richness compared to intact forest plots (Dunnett's post-hoc test *p = *0.009). In 100-ha plots, time since fragmentation was also significant in determining adjusted species richness (F_3,10_ = 4.493, *p = *0.030, Figure 2C), as fragmented forest plots two years after fragmentation had significantly more species than intact forest plots (Dunnett's post-hoc test *p = *0.018).

### Temporal and Spatial Variation in Adjusted and Rarefied Species Richness

**Figure 3:**
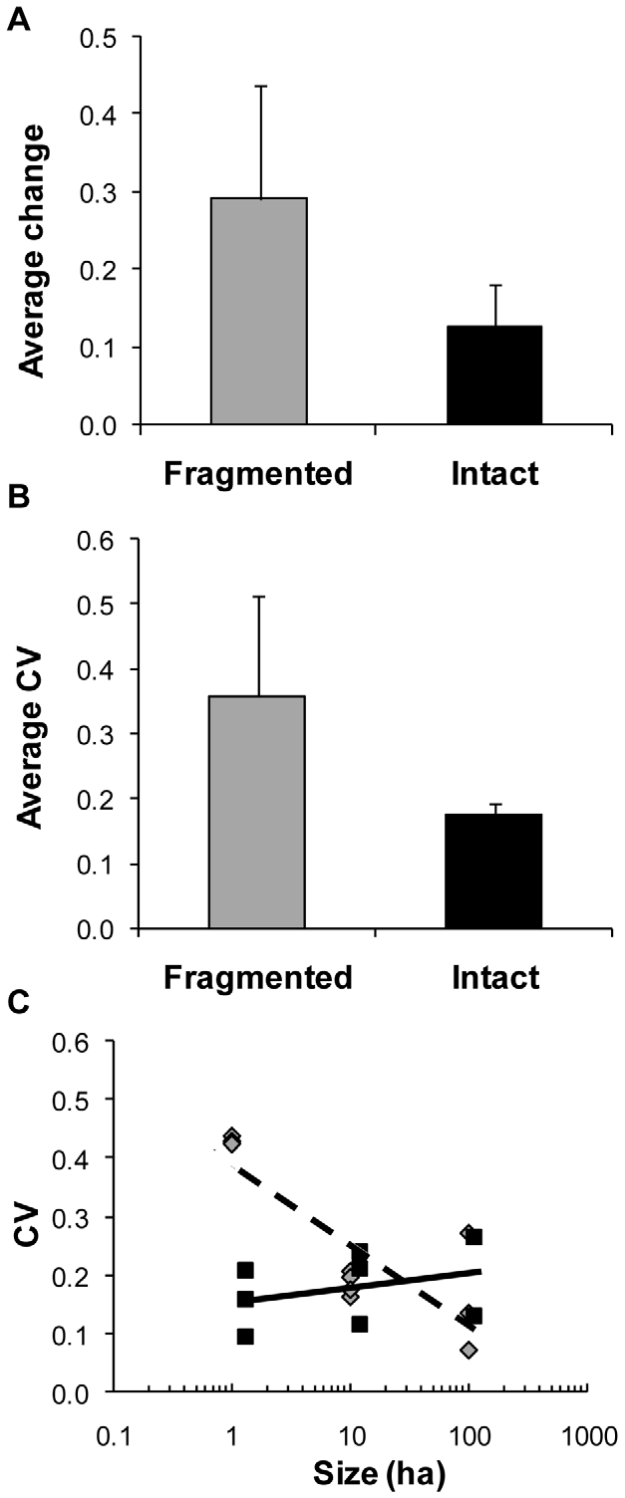
Spatial and temporal variation in species richness is higher in fragmented forest plots. Variation in adjusted species richness for fragmented and intact forest plots for (A) average change within plots over time (temporal variation) and (B) average variation between plots at a given point in time (spatial variation). (C) A regression of average *cv* between plots of a given fragmentation status (fragmented or intact) at a specific point in time against log-transformed plot size shows that fragmented (gray diamonds, dashed regression line) 1-ha plots have greater variation than other plot types and sizes. Intact plots are represented by solid squares and a solid regression line. Error bars represent standard deviation.

Temporal variation in adjusted species richness was significantly greater in fragmented forest plots relative to intact forest plots (*t*_14_ = 4.029, *p = *0.001, Figure 3A). Fragmented forest plots also had significantly higher spatial variation in adjusted species richness (*t*_5_ = 2.910, *p = *0.032, Figure 3B). Increased spatial variation was only evident in 1-ha fragmented forest plots, which were highly variable in their numbers of species within each time period (*t = *4.150, *p* = 0.032, *R^2^* = 0.682, *n = *10, *b* = −0.059, 95% CI: −0.026–−0.092, Figure 3C). There was no effect of plot size on spatial variation among intact forest plots (*t = *0.800, *p* = 0.454, *R^2^* = 0.097, *n = 8*, *b* = 0.010, 95% CI: −0.021–0.041, Figure 3C). In similar analyses of rarefied species richness, there was no longer a difference in temporal or spatial variation between intact and fragmented plots (temporal, *t*_18_ = 1.333, *p = *0.199; spatial, *t*_8_ = 1.598, *p = *0.149).

### Community Composition

**Figure 4:**
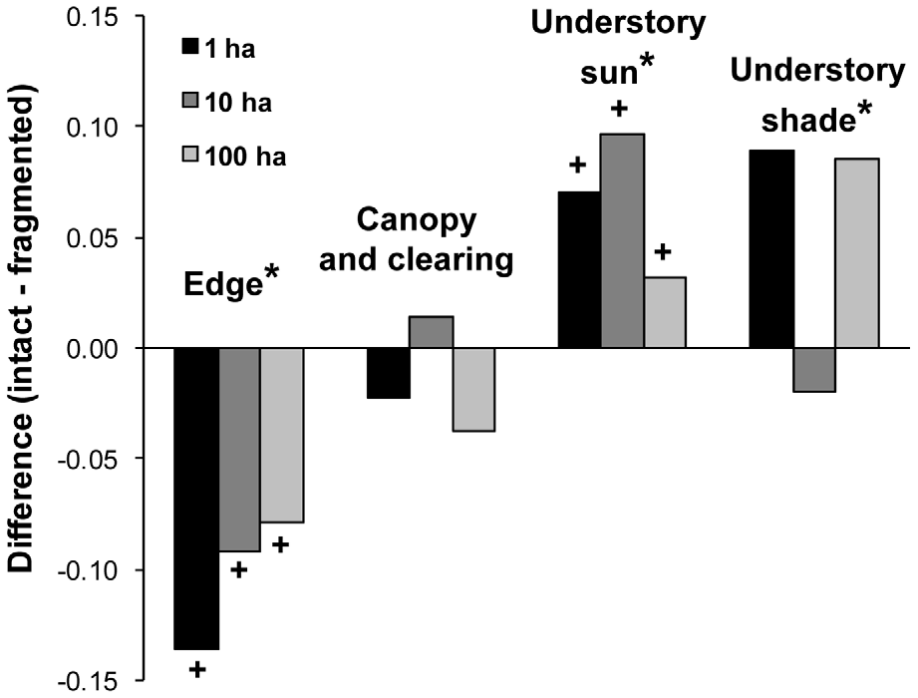
Differences in community composition between fragmented and intact forest plots. For each habitat association, differences were taken as average proportion in intact plots–average proportion in fragmented plots. Therefore, negative values represent cases where a habitat association had a higher average proportion in fragmented forest plots relative to intact forest plots. Asterisks denote habitat associations for which fragmentation status was significant and plus signs denote associations for which size was significant (see Table 1).

Fragmented forest plots of all sizes harbored different butterfly communities relative to intact forest plots (Figure 4, Table 1). Relative to intact forest plots, the proportion of edge species in fragmented forest plots significantly increased and the proportion of understory shade and understory sun species in fragmented forest plots decreased. Species associated with canopies and clearings had roughly the same percent composition in intact and fragmented forest plots. The interaction between plot size and fragmentation status (fragmented vs. intact) was not significant for any of the habitat associations, so it was removed from the analysis.

**Table 1:** ANOVA results for the effect of size and fragmentation status (fragmented *vs*. intact) on each of the four habitat associations.

Habitat association/variable		df	F	*p*
Edge (*r^2^* = 0.877)		2,19	68.022	<0.001
	ln(size)	1	4.493	0.047
	fragmentation status	1	131.550	<0.001
Canopy and clearing (*r^2^* = 0.121)		2,19	1.307	0.294
	ln(size)	1	1.856	0.189
	fragmentation status	1	0.758	0.395
Understory sun (*r^2^* = 0.721)		2,19	24.507	<0.001
	ln(size)	1	5.852	0.026
	fragmentation status	1	43.163	<0.001
Understory shade (*r^2^* = 0.376)		2,19	5.733	0.012
	ln(size)	1	1.918	0.182
	fragmentation status	1	9.548	0.006

## Discussion

Our results show that tropical forest fragmentation increased the temporal and spatial variability of species richness adjusted for survey duration in butterfly communities. We examined three mechanisms to explain this higher variability. First, the variability in adjusted species richness was not associated with a change in average species richness between fragmented and intact forests, but was associated with a change in community composition. Second, variability was associated with changes in butterfly community composition. Butterfly communities in fragmented and intact forest had similar proportions of species associated with high light environments, such as canopies and clearings. However, fragmentation increased the proportion of edge species and decreased the proportion of shade-dwelling species associated with closed canopy forest. Third, when we accounted for abundance in estimates of rarefied species richness, using the limited data we had available (indices of abundance), fragmented forest plots were no more spatially or temporally variable than intact forest plots. Combined, these results suggest that the increased variability in butterfly species richness in forest fragments may be explained by changes in community composition and butterfly abundance. Consequently, the natural variability of butterflies was not a buffer against the effects of fragmentation on community dynamics [12].

The response of butterfly species richness to habitat fragmentation and plot size demonstrates how fragmentation effects can be obscured by opposing responses of different species groups (Figure 1). Small fragments had lower species richness than similar size areas of intact forest. This result is consistent with small fragments being unable to support butterfly species that are forest specialists. On the other hand, large fragmented forest plots actually had more butterfly species than similar size plots in intact forest. This is because large fragments had both forest specialists and edge species. These results further support how species traits, in this case habitat specialization, are critical to understanding fragmentation's effects.

Our results show evidence that hyperdynamism in forest fragments may be caused by changes in abundance. One cause for changes in abundance may be changes in resource availability [27]. For species that exploit resources in the matrix or at habitat edges, fragmentation may lead to outbreaks, providing greater source populations for colonization events. Fragmentation can also lead to the homogenization of plant communities, as has been shown in the Biological Dynamics of Forest Fragments Project (BDFFP) for tree seedlings, palms, herbs, and lianas [9]. Ultimately, the consequences of rapid positive and negative population fluctuations of butterflies would contribute to increased temporal and spatial fluctuations of species richness. Our imprecise abundance data allow only limited interpretations, but a future avenue of research would be to investigate how fragmentation changes temporal dynamics of butterfly populations in tropical forests. Regardless of whether abundance proves the most important mechanism explaining changes in species richness, responses of both measures–richness and abundance–independently provide important information for biodiversity conservation in fragmented landscapes.

The change in community composition may also contribute to hyperdynamism in fragmented forests, as the community could have shifted toward species or species groups that are more variable in abundance. The diversity of butterfly species associated with closed canopy forest (understory shade species) and understory sun species were the most severely reduced by fragmentation. Although average adjusted species richness did not differ between fragmented and intact forests, the proportion of understory sun and shade species significantly declined in fragmented forest plots, indicating local extinctions of these butterflies. The decline in shade species likely reflects a decline in their available habitat in proportion to fragment size. Species associated with high light environments, such as canopy and clearing species, would be less affected by changes in habitat structure and microclimate near habitat edges because the new habitat more closely resembles their natural habitat.

Uncontrolled aspects of the experimental design could contribute to our findings of higher variability of adjusted species richness in forest fragments. Variation in landscape features, including the distance to intact forest and the type of matrix vegetation, could contribute to the higher spatial variability of species richness in fragmented forest plots. Although these are other interesting consequence of fragmentation, the differences we observed are not confounded by them, as our measure of temporal variability compared the average change *within* plots over time.

The logistical and statistical hurdles faced in this study are representative of many large-scale, long-term studies that analyze multiple species. Ideally but unrealistically, all plots would have been completely surveyed multiple times per year, with precise measures of species abundance for monitoring population trends. One alternative, focusing intensively on just a few charismatic or rare species, may give misleading information about overall community dynamics, particularly given the number of rare species in tropical forests. By grouping species based on life history traits, feeding guilds, or habitat associations, we can use more targeted methods to assess the effects of fragmentation. In turn, these data could inform conservation strategies designed to protect the most vulnerable species.

Our findings of equal adjusted species richness in fragmented and intact forest plots are consistent with those from a study of trees in the same experiment [13]. However, studies of other animals in this system found lower species richness of birds [28], [29] and higher species richness of small mammals and frogs in fragmented forest relative to intact forest [30], [31]. Our results are consistent with findings in the Atlantic Forest of Brazil, where habitat fragmentation did not impact species richness of fruit-feeding butterflies, but did alter community composition [32]. We recognize that feeding guild or habitat association could explain only part of the story as to which species are more vulnerable to fragmentation. Yet, knowledge about the susceptibility of species groups can be used to guide future studies of ecological traits that may influence species vulnerability to extinction in fragmented environments [33]. For butterflies, these include degree of specialization [34] and body size [35].

Ecological theory provides compelling evidence that the spatial structure of landscapes can affect the stability of community dynamics [36]. Our results provide some empirical support for this theory and point to interesting new avenues of research on forest fragmentation and butterfly communities. Given our finding that butterfly abundance and habitat association may be driving variation in species richness in fragmented landscapes, more attention should be focused on long-term population stability within fragments. In our study, this would mean a several year investigation of the population sizes of key focal species that are spread among different habitat associations. Although these intensive data would provide stronger, mechanistic tests of our findings, a focus on more limited surveys of species richness was still able to elucidate broad patterns in the effects of forest fragmentation.

Two main conservation recommendations for monitoring insects in fragmented habitats come from our findings that butterfly communities display characteristics of hyperdynamism. First, long time-series of community data are critical to understanding the effects of fragmentation. Increased spatial and temporal variation of adjusted species richness in fragmented plots was not associated with a change in overall species richness. However, a survey at any given time after fragmentation could have found either higher or lower species richness in fragmented plots, relative to intact plots, simply by chance. By looking at changes in richness over time, we found that fragmentation affected the dynamics of species richness, as opposed to static values of species richness. Second, given limited time and resources, monitoring based on community composition, rather than species richness, provides quick feedback to conservation planners on the effects of fragmentation. Analyses based on percent composition may reduce the effect of inaccurate species identification and help in cases where there are many rare species in an extremely diverse fauna.

## Supporting Information

Text S1 Supporting text.
(0.03 MB DOC)

Figure S1 Schematic of butterfly surveys used in the analyses. For each survey, the date and duration of the survey (in hours) is listed. The year a plot was fragmented (top of figure) is outlined in black.
(0.57 MB TIF)

Figure S2 The effect of seasonality on species richness. The residuals from the regression of combined species richness against the number of months between surveys was not significant for intact forest plots (solid squares, solid regression line), but was significant for fragmented plots (gray diamonds, dashed regression line).
(0.14 MB TIF)

Figure S3 Regression of species richness against survey hours for intact forest plots. Surveys are coded by plot size for visual emphasis only, as plot size was not a significant variable in determining species richness.
(0.14 MB TIF)

Figure S4 Regression of species richness against survey hours for fragmented forest plots. (A) All plots combined. Surveys are coded by plot size for visual emphasis only. (B) The 1 ha regression was not significant, but the 10 ha (C) and 100 ha (D) regressions were significant.
(0.19 MB TIF)

Table S1 ANOVA results for species richness in intact forest plots with significant variables for the effects test bolded.
(0.01 MB PDF)

Table S2 ANOVA results for species richness in fragmented forest plots with significant variables for the effects test bolded (*p*<0.100).
(0.04 MB PDF)
