# Supplementary material for: Does Tropical Forest Fragmentation Increase Long-Term Variability of Butterfly Communities?
Source: PLoS One. 2010 Mar 10;5(3):e9534. doi: 10.1371/journal.pone.0009534 (PMC2835745; doi:10.1371/journal.pone.0009534)
Supplement: Text S1 — Supporting text. (0.03 MB DOC) [file pone.0009534.s001.doc]

**SUPPORTING TEXT**

The turnover of species richness within a given plot over the course of a calendar year prevented us from combining data from multiple surveys within a year (for example, when a plot was surveyed in both February and November). We tested for a seasonality effect by identifying all cases where a plot was surveyed multiple times within a year, and calculated total species richness for that plot within that year. We then took the values of total species richness and regressed them against observer hours (survey effort). From this analysis, we plotted the residuals against the number of months between the first and last survey of that year, where 0 indicates that the surveys were conducted in the same month.

If seasonality is an issue, we would expect to find larger, positive residuals for cases where surveys separated by many months were combined. We found a seasonality effect for fragmented, but not intact, forest plots (Figure S2). A regression of the residuals against the number of months was not significant for intact forest plots (*t =* 0.18, *p* = 0.856, *R^2^* = 0.002, *n =* 18*,* *b* = -0.358). The regression was significant for fragmented plots (*t =*6.08*,* *p* <0.001, *R^2^* = 0.804, *n =* 11) and the slope was positive (*b* = 9.228).
