# Supplementary figures and images for: Does Tropical Forest Fragmentation Increase Long-Term Variability of Butterfly Communities?
Source: PLoS One. 2010 Mar 10;5(3):e9534. doi: 10.1371/journal.pone.0009534 (PMC2835745; doi:10.1371/journal.pone.0009534)

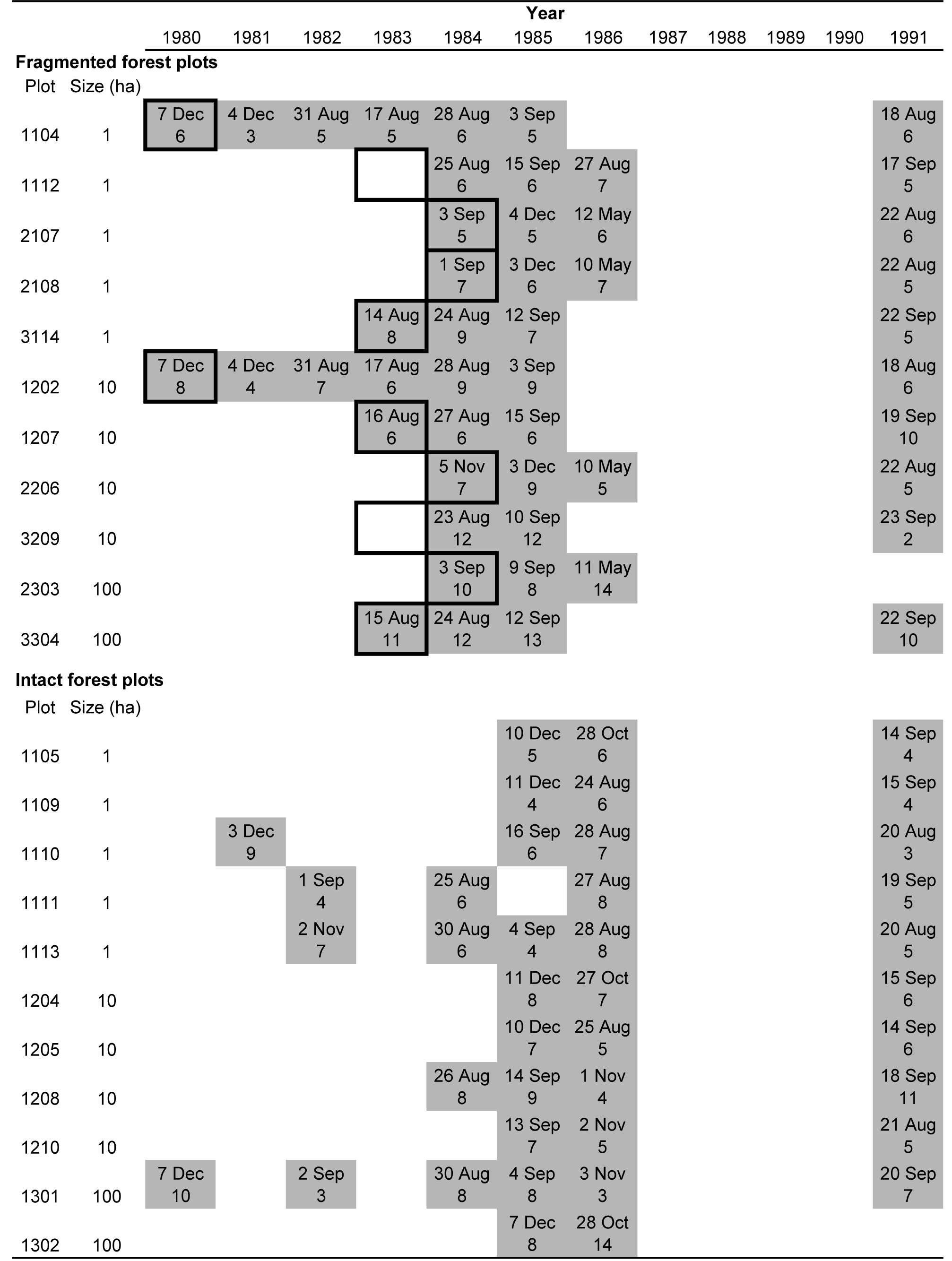

Supplement: Figure S1 — Schematic of butterfly surveys used in the analyses. For each survey, the date and duration of the survey (in hours) is listed. The year a plot was fragmented (top of figure) is outlined in black. (0.57 MB TIF) [file pone.0009534.s002.tif]

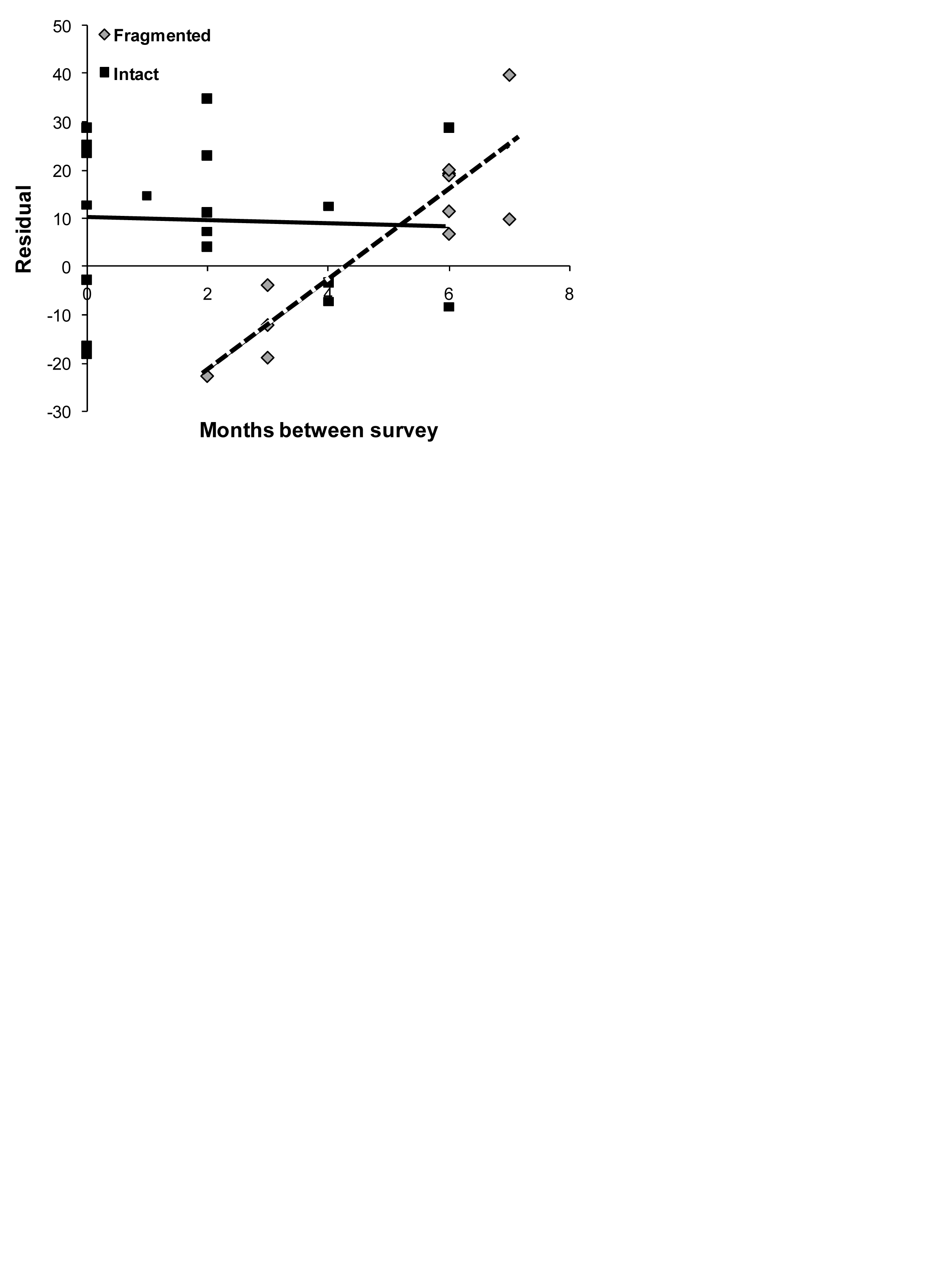

Supplement: Figure S2 — The effect of seasonality on species richness. The residuals from the regression of combined species richness against the number of months between surveys was not significant for intact forest plots (solid squares, solid regression line), but was significant for fragmented plots (gray diamonds, dashed regression line). (0.14 MB TIF) [file pone.0009534.s003.tif]

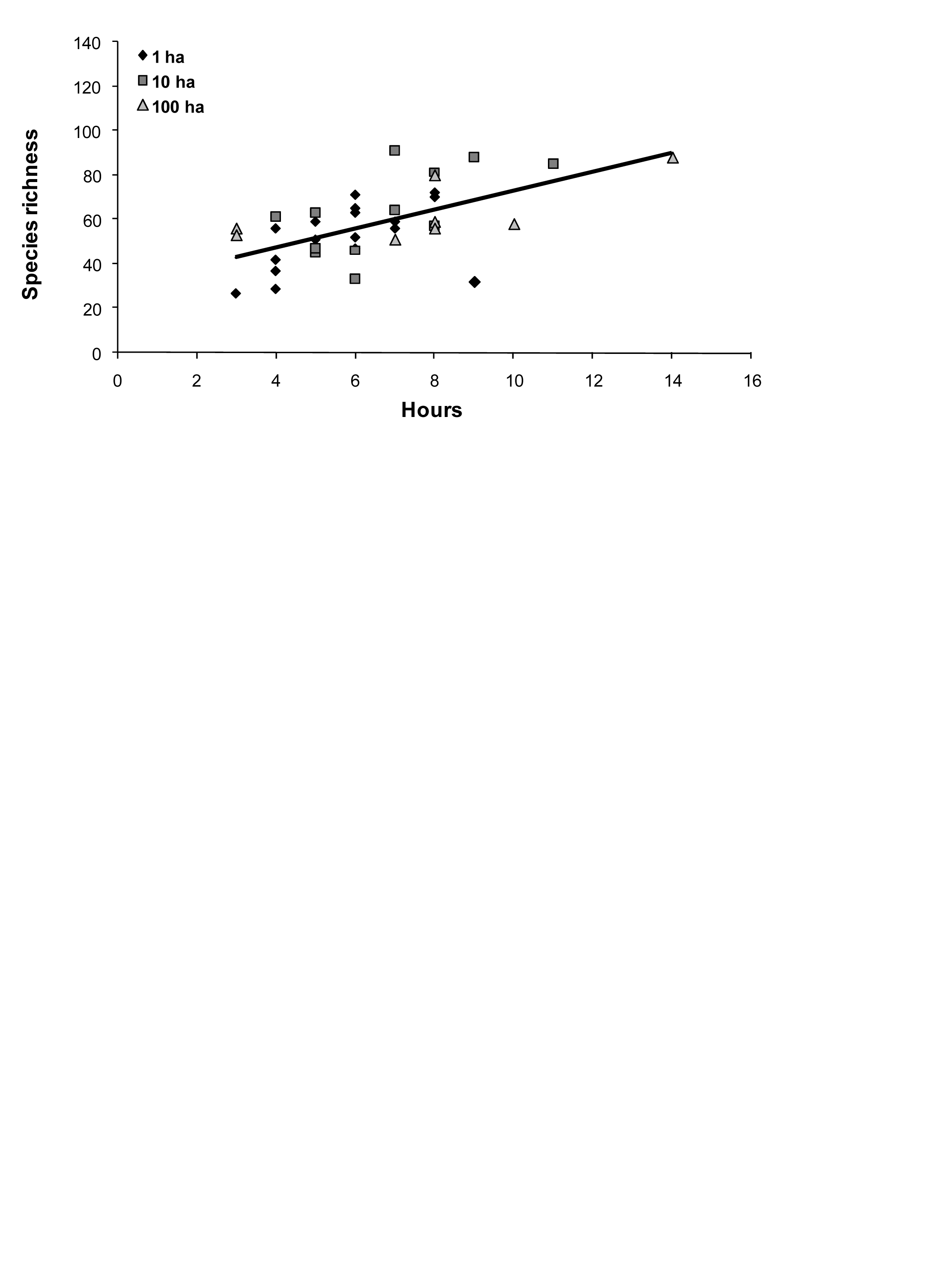

Supplement: Figure S3 — Regression of species richness against survey hours for intact forest plots. Surveys are coded by plot size for visual emphasis only, as plot size was not a significant variable in determining species richness. (0.14 MB TIF) [file pone.0009534.s004.tif]

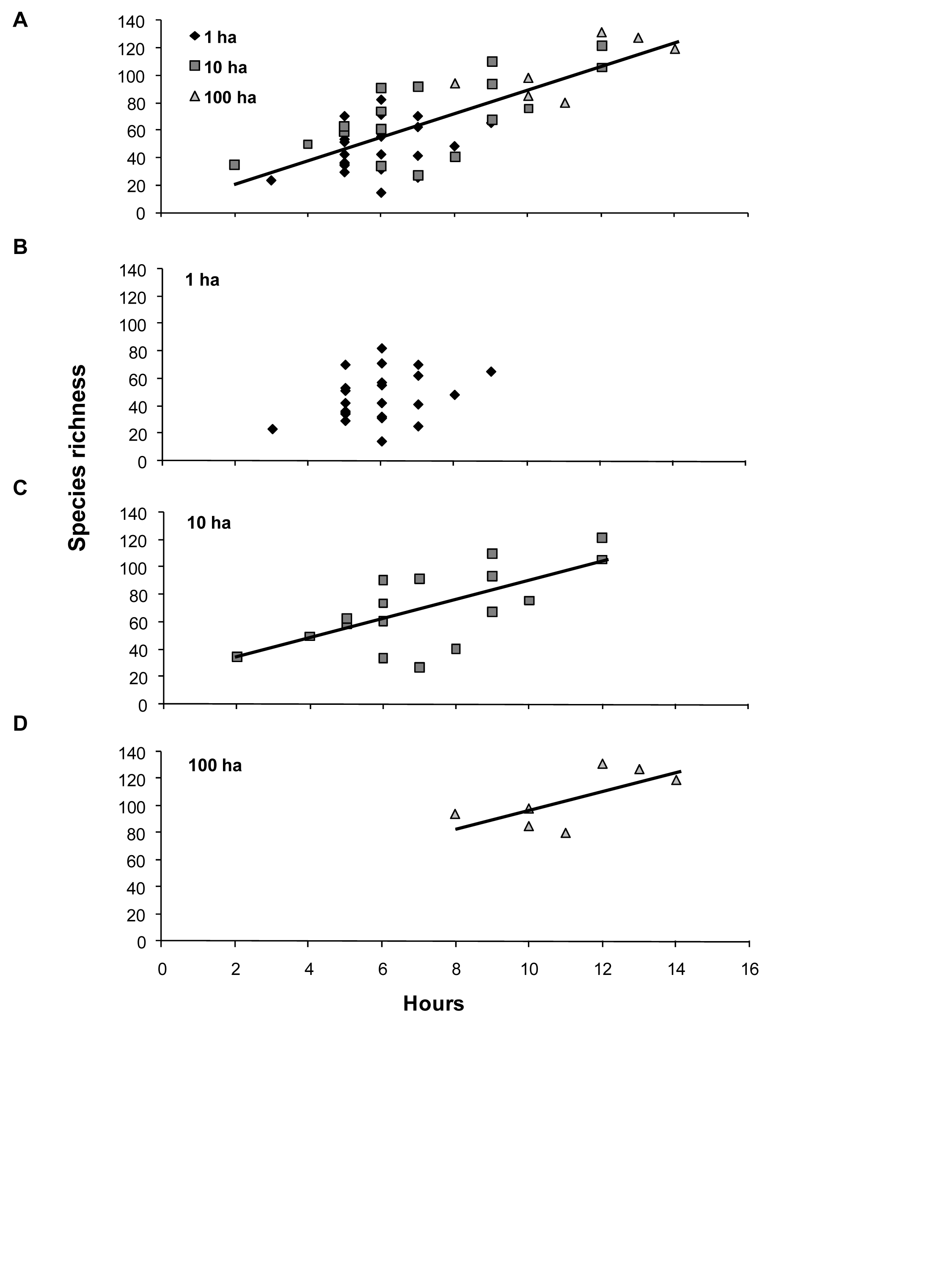

Supplement: Figure S4 — Regression of species richness against survey hours for fragmented forest plots. (A) All plots combined. Surveys are coded by plot size for visual emphasis only. (B) The 1 ha regression was not significant, but the 10 ha (C) and 100 ha (D) regressions were significant. (0.19 MB TIF) [file pone.0009534.s005.tif]
