# Supplementary material for: Does Tropical Forest Fragmentation Increase Long-Term Variability of Butterfly Communities?
Source: PLoS One. 2010 Mar 10;5(3):e9534. doi: 10.1371/journal.pone.0009534 (PMC2835745; doi:10.1371/journal.pone.0009534)
Supplement: Table S1 — ANOVA results for species richness in intact forest plots with significant variables for the effects test bolded. (0.01 MB PDF) [file pone.0009534.s006.pdf]

Table S1

	Whole Model			Effects Test (p-Value)			
	**F**	***p***	**r^2**	**hours**	**plot**	**size**	**year**
plot, hours, year	3.325	0.005	0.596	**0.014**	0.193		0.240
size, hours, year	7.525	<0.001	0.385	**<0.001**		0.384	0.581
hours, plot	3.441	0.004	0.575	**0.007**	0.243		
hours	21.956	<0.001	0.366	**<0.001**			
