# Supplementary material for: Does Tropical Forest Fragmentation Increase Long-Term Variability of Butterfly Communities?
Source: PLoS One. 2010 Mar 10;5(3):e9534. doi: 10.1371/journal.pone.0009534 (PMC2835745; doi:10.1371/journal.pone.0009534)
Supplement: Table S2 — ANOVA results for species richness in fragmented forest plots with significant variables for the effects test bolded (p<0.100). (0.04 MB PDF) [file pone.0009534.s007.pdf]

## Table S2

| | Whole Model | | | Effects Test (p-Value) | | | | |
|---|---|---|---|---|---|---|---|---|
| | **F** | ***p*** | **$r^2$** | **hours** | **plot** | **size** | **year** | **ysf** |
| **All sizes** | | | | | | | | |
| plot, hours, year | 7.631 | <0.001 | 0.723 | **<0.001** | **0.094** | | 0.362 | |
| plot, hours, ysf | 7.631 | <0.001 | 0.723 | **<0.001** | 0.145 | | | 0.362 |
| plot, hours | 8.281 | <0.001 | 0.717 | **<0.001** | 0.105 | | | |
| size, hours, ysf | 27.180 | <0.001 | 0.658 | **<0.001** | | **0.007** | | 0.189 |
| size, hours, year | 26.580 | <0.001 | 0.645 | **<0.001** | | **0.007** | 0.782 | |
| size, hours | 40.665 | | 0.644 | **<0.001** | | **0.006** | | |
| hours | 63.133 | <0.001 | 0.579 | **<0.001** | | | | |
| **1 ha** | | | | | | | | |
| plot, hour, year | 1.675 | 0.191 | 0.386 | **0.094** | 0.180 | | 0.650 | |
| plot, hour, ysf | 1.675 | 0.191 | 0.386 | **0.094** | 0.210 | | 0.650 | |
| plot, hours | 2.063 | 0.121 | 0.378 | **0.097** | 0.171 | | | |
| hours | 2.611 | 0.121 | 0.111 | 0.121 | | | | |
| **10 ha** | | | | | | | | |
| plot, hour, year | 3.007 | 0.055 | 0.556 | **0.007** | 0.592 | | 0.355 | |
| plot, hour, ysf | 3.007 | 0.055 | 0.556 | **0.007** | 0.734 | | 0.355 | |
| plot, hours | 3.547 | 0.260 | 0.522 | **0.008** | 0.619 | | | |
| hours | 13.320 | 0.002 | 0.454 | **0.002** | | | | |
| **100 ha** | | | | | | | | |
| plot, hour, year | 0.988 | 0.504 | 0.497 | 0.205 | 0.874 | | 0.753 | |
| plot, hour, ysf | 0.988 | 0.504 | 0.497 | 0.205 | 0.927 | | 0.753 | |
| plot, hours | 1.824 | 0.274 | 0.477 | 0.129 | 0.880 | | | |
| hours | 4.387 | 0.090 | 0.467 | **0.090** | | | | |
